# Numerical Simulation of CO_2_ Flooding of Coalbed Methane Considering the Fluid-Solid Coupling Effect

**DOI:** 10.1371/journal.pone.0152066

**Published:** 2016-03-31

**Authors:** Jianjun Liu, Guang Li, Yue Zhang

**Affiliations:** 1 School of Geoscience and Technology, Southwest Petroleum University, Chengdu, China; 2 State Key Laboratory of Oil and Gas Reservoir Geology and Exploitation, Southwest Petroleum University, Chengdu, China; 3 Jidong Oilfield Company, PetroChina, Tangshan, Hebei, China; University of Akron, UNITED STATES

## Abstract

CO_2_ flooding of coalbed methane (CO_2_-ECBM) not only stores CO_2_ underground and reduces greenhouse gas emissions but also enhances the gas production ratio. This coupled process involves multi-phase fluid flow and coal-rock deformation, as well as processes such as competitive gas adsorption and diffusion from the coal matrix into fractures. A dual-porosity medium that consists of a matrix and fractures was built to simulate the flooding process, and a mathematical model was used to consider the competitive adsorption, diffusion and seepage processes and the interaction between flow and deformation. Due to the effects of the initial pressure and the differences in pressure variation during the production process, permeability changes caused by matrix shrinkage were spatially variable in the reservoir. The maximum value of permeability appeared near the production well, and the degree of rebound decreased with increasing distance from the production well.

## Introduction

The porosity and permeability of fracture systems in coalbed methane reservoirs are influenced by effective stress and gas adsorption-desorption. In 1987, Gray put forward that when the coalbed methane desorbs, the coal matrix shrinks, which can cause crack expansion and permeability increases [1]. In 1998, Mavor used observational data from the San Juan Basin to prove the coal matrix shrinkage hypothesis [2]. In the same year, Palmer and Mansoori derived the permeability calculation formula (P&M formula), which considers the effects of effective stress and matrix shrinkage on permeability. Based on this formula, they studied the actual production process of the San Juan Basin and successfully explained the “gas production rebound” phenomenon [3]. At present, many scholars have performed numerous studies on experimental and theoretical aspects. Jessen [4] reported a mixed gas injection mechanism based on research on the different effects associated with enhancing recovery efficiency using injected CO_2_, N_2_ and mixed gases. Karacan [5] found that adsorption and swelling phenomena in coal were heterogeneous and different parts of a coal sample behave differently. Gensterblum [6] reported that there were three relationships between coal swelling and the amount of CO_2_ adsorbed by coal and that coal swelling was not affected at pressures below a few atmospheres. An [7] carried out an experimental and numerical investigation on anisotropic permeability of coal and evaluated the effects of the anisotropic permeability variation on CO_2_-ECBM. Kumar [8] investigated the evolution of permeability heterogeneity during CO_2_-ECBM. Alexej [9] analyzed the effect of moisture on sorption capacity for coals of different rank and the competitive CO_2_/CH_4_ sorption behavior in binary gas mixtures by using laboratory experiment method. Massarotto [10] researched the changes in reservoir properties from injection of supercritical CO_2_ into coal seams. In addition, deformations induced by adsorption were studied by the researchers [11–14]. However, those studies of CO_2_ flooding of coalbed methane mostly focus on competitive adsorption between CO_2_ and CH_4_, gas-water two-phase flow and the effect caused by coal or rock deformation on permeability. Very little research has been conducted on the dynamic evolution of permeability during CO_2_ injection.

In this paper, based on the existing theory and test results [15–20], a mathematical model considered competitive adsorption, diffusion and seepage process and the interaction between flow and deformation was established, using software simulated the production situation of CO_2_ flooding, emphasis analyses the dynamic evolution of permeability during the process of CO_2_ flooding.

## Mathematical Model of CO_2_ Flooding of Coalbed Methane

Model assumptions: Both the coalbed methane and water flow under Darcy flow, and the two-phase fluid flow can be expressed as follows:

$$\frac{\partial }{\partial x}\left[\frac{k_{rg}k_{x}}{B_{g}\mu_{g}}\left(\frac{\partial p_{g}}{\partial x}-\rho_{g}g\frac{\partial D}{\partial x}\right)\right]+\frac{\partial }{\partial y}\left[\frac{k_{rg}k_{y}}{B_{g}\mu_{g}}\left(\frac{\partial p_{g}}{\partial y}-\rho_{g}g\frac{\partial D}{\partial y}\right)\right]+\frac{\partial }{\partial z}\left[\frac{k_{rg}k_{z}}{B_{g}\mu_{g}}\left(\frac{\partial p_{g}}{\partial z}-\rho_{g}g\frac{\partial D}{\partial z}\right)\right]+q_{f}-q_{g}=\frac{\partial }{\partial t}\left(\phi \rho_{g}S_{g}\right)$$ (1)

$$\frac{\partial }{\partial x}\left[\frac{k_{rw}k_{x}}{B_{w}\mu_{w}}\left(\frac{\partial p_{w}}{\partial x}-\rho_{w}g\frac{\partial D}{\partial x}\right)\right]+\frac{\partial }{\partial y}\left[\frac{k_{rw}k_{y}}{B_{w}\mu_{w}}\left(\frac{\partial p_{w}}{\partial y}-\rho_{w}g\frac{\partial D}{\partial y}\right)\right]+\frac{\partial }{\partial z}\left[\frac{k_{rw}k_{z}}{B_{w}\mu_{w}}\left(\frac{\partial p_{w}}{\partial z}-\rho_{w}g\frac{\partial D}{\partial z}\right)\right]-q_{w}=\frac{\partial }{\partial t}\left(\phi \rho_{w}S_{w}\right)$$ (2)

where *k*_*rg*_ is the relative gas permeability; *k*_*rw*_ is the relative water permeability; *k*_x,_ *k*_y_, and *k*_z_ are the absolute permeabilities in the X, Y, and Z directions, respectively; *ρ*_*g*_ is the gas density; *ρ*_*w*_ is the water density; g is the acceleration of gravity, m/s^2^; *P*_*g*_ is the gas pressure, MPa; *P*_*w*_ is the water pressure, MPa; *S*_*g*_ is the gas saturation; *S*_*w*_ is the water saturation; *ϕ* is porosity; *D* is standard height, m; *μ*_*g*_ is the viscosity of the gas; *μ*_*w*_ is the viscosity of the water; *B*_*g*_ is the formation volume factor of the gas; *B*_*w*_ is the formation volume factor of the water; *q*_*f*_ is the amount of gas in the fracture due to the diffusion effect; *q*_*g*_ is the source term of the gas, m^3^/d; and *q*_*w*_ is the source term of the water, m^3^/d.

Permeability in coals is a function of effective stress and matrix shrinkage. The calculation uses the P&M equation (Palmer and Mansoori 1998 [3]), which is expressed as follows:

$$\frac{\phi_{f}}{\phi_{f0}}=1+C_{f}\left(P-P_{i}\right)+\xi_{L}\left(1-\frac{K}{M}\right)\left(\frac{P_{i}}{P_{i}+P_{L}}-\frac{P}{P+P_{L}}\right)$$ (3)

In Eq (3), the central part indicates the influence of stress change (cleat deformation) on porosity, the latter part indicates the influence of matrix shrinkage on porosity, when considers only cleat deformation, the expression can be expressed as Eq (4):

$$\frac{\phi_{f}}{\phi_{f0}}=1+C_{f}\left(P-P_{i}\right)$$ (4)

$$\frac{k_{f}}{k_{f0}}={\left(\frac{\phi_{f}}{\phi_{f0}}\right)}^{3}$$ (5)

where $C_{f}=\frac{1}{\phi_{0}M}$; $\frac{K}{M}=\frac{1}{3}\left(\frac{1+\mu }{1-\mu }\right)$; $M=E\frac{1-\mu }{\left(1+\mu \right)\left(1-2\mu \right)}$

*ϕ*_*f*_ is the fracture porosity at pressure p; *ϕ*_*f*0_ is the initial natural fracture porosity at a given pressure; *C*_*f*_ is the pore volume compression coefficient, 1/kPa; *ξ*_*L*_ is the strain at infinite pressure; *K* is the bulk modulus, kPa; *M* is the axial modulus, kPa; *P*_*i*_ is the initial pressure, kPa; *K*_*f0*_ is the initial permeability, *E* is the elasticity modulus, kPa; and *μ* is Poisson's ratio; *ϕ*_0_ is the initial porosity, *C*_*f*_ is rock compressibility.

Assuming that the absorption behaviors of CH_4_ and CO_2_ in coal follow the rule of Langmuir, the amount of adsorbed gas can be expressed as follows:

$$V_{i}=\frac{{\left(V_{m}\right)}_{i}b_{i}p_{i}}{1+\sum_{j=1}^{n}b_{j}p_{j}}$$ (6)

where *(V*_*m*_*)*_*i*_ is the adsorption constant of the pure species gas i, cm^3^/g; *b*_*i*_ is the pressure-constant of the pure species gas i, 1/MPa; and *p*_*i*_ is the partial pressure of gas component i, MPa.

Coalbed methane diffuses from the matrix to fractures following Fick’s law:

$$\frac{\partial C}{\partial t}=\frac{\partial^{2}C}{\partial X^{2}}$$ (7)

where *C* is the gas concentration, mol/m, and *X* is the distance of gas diffusion, m.

The convection diffusion equation for gas can be written as follows:

$$\frac{\partial }{\partial t}\left(\phi \left(p\right)C\right)+\nabla \left(-D\left(\mu \right)+C\mu \right)=0$$ (8)

where *u* is the pore velocity of fluid, m/s, and D is the tensor diffusion, m^2^/s.

The capillary pressure equation and the saturation equation can be written, respectively as follows:

$$P_{c}=P_{g}-P_{w}$$ (9)

$$S_{g}+S_{w}=1$$ (10)

where *P*_*c*_ is the capillary pressure, Pa.

## Numerical Simulation of CO_2_ Flooding of Coalbed Methane

**Fig 1:**
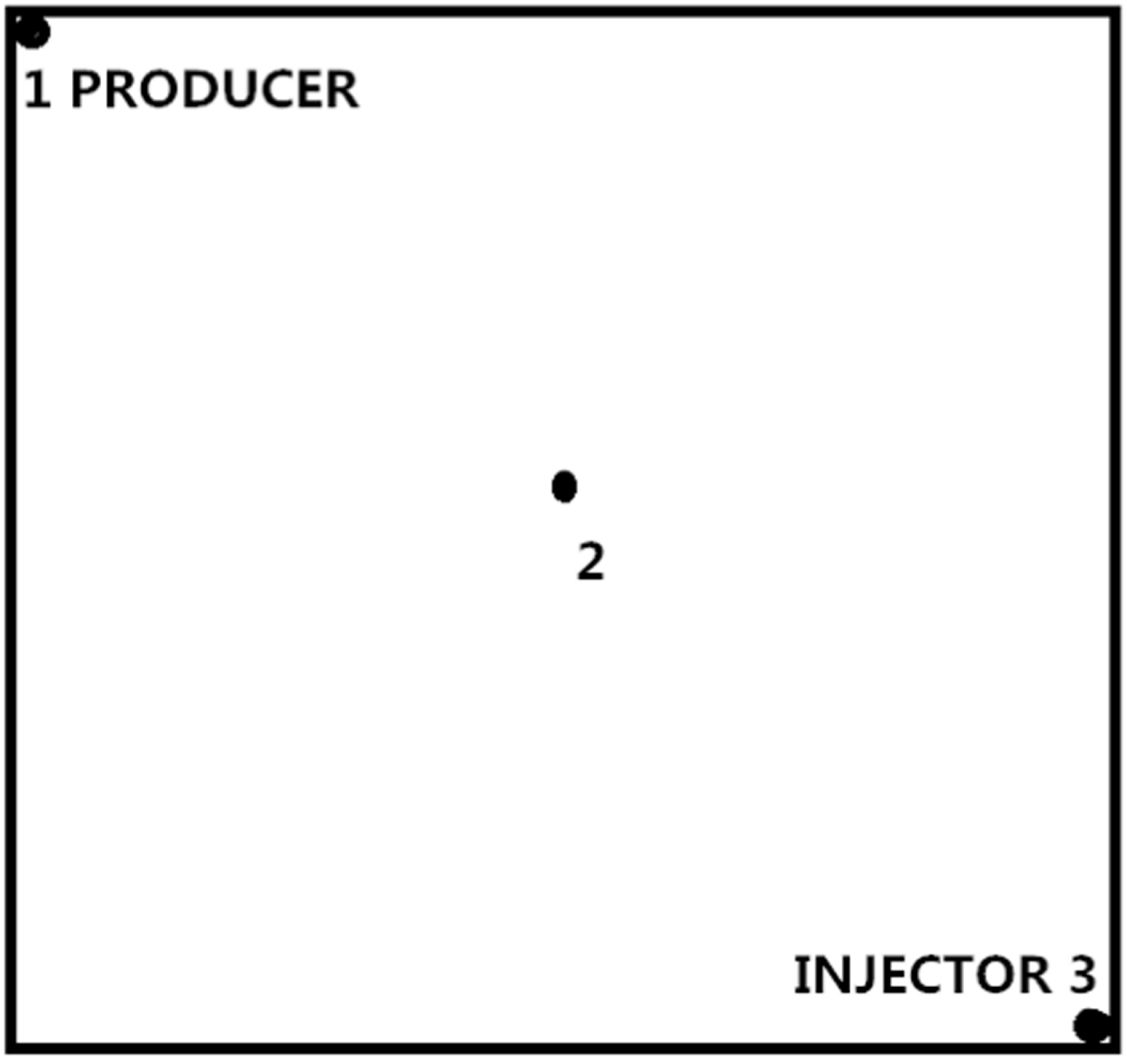
Well location.

A commercial reservoir simulator was used in this study (CMG-GEM, 2012). The well location distribution is shown in Fig 1. The model used closed boundaries and the change of temperature was not considered in our study. CO_2_ was injected using a constant rate of 2000 m^3^/d. (at surface condition) for a period of 1940 days. Relevant parameters are provided in Table 1.

**Table 1:** Selected parameters.

Parameters	Value	Parameters	Value
**Gridding**	40*40*1	**Reservoir temperature (°C)**	45
**Grid spacing (m)**	5 5 9	**Reservoir pressure (MPa)**	7.65
**Young’s modulus of elasticity (kPa)**	3000000	**Top depth (m)**	900
**Matrix porosity (%)**	0.5	**Cleat porosity (%)**	0.1
**Poisson ratio**	0.4	**CO**_**2**_**/CH**_**4**_ **Langmuir pressure (kPa)**	1090/350
**CO**_**2**_**/CH**_**4**_ **Diffusion value (d)**	100/100	**Cleat permeability (mD)**	4.0
**Coal compression coefficient (kPa**^**-1**^)	1.45E-7	**Coal density (kg/m**^**3**^)	1400
**CO**_**2**_ **maximum adsorption capacity by unit mass (mol/kg)**	1.0	**CH**_**4**_ **maximum adsorption capacity by unit mass (mol/kg)**	0.5

**Fig 2:**
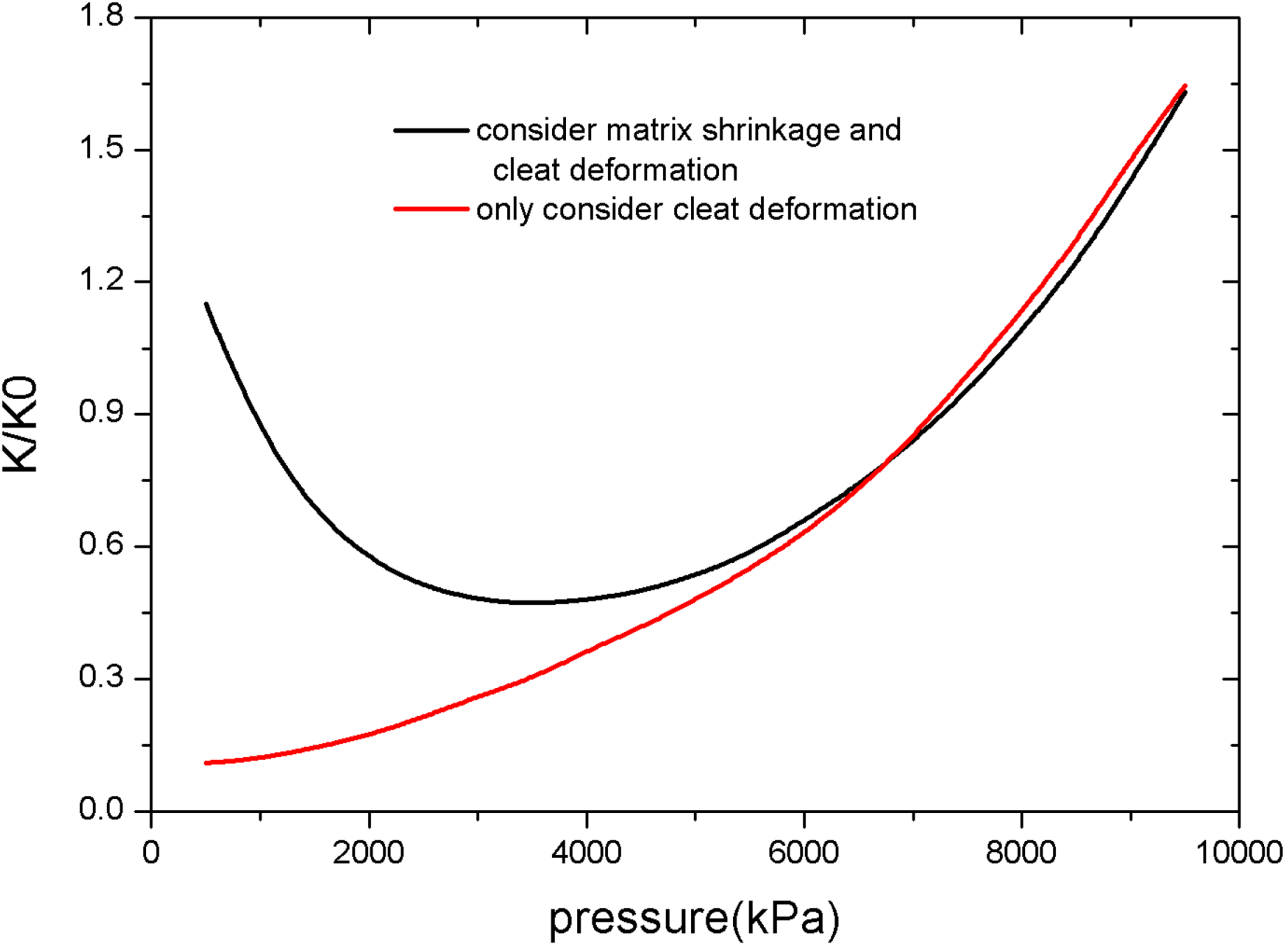
Permeability models used in the simulation.

Fig 2 compares the two permeability models used in the simulation. A significant difference in permeability changes is present between the two models. The effect on permeability caused by matrix shrinkage appears when the reservoir pressure reaches a low level (approximately 3.5 MPa), and becomes more significant with further drops in reservoir pressure.

**Fig 3:**
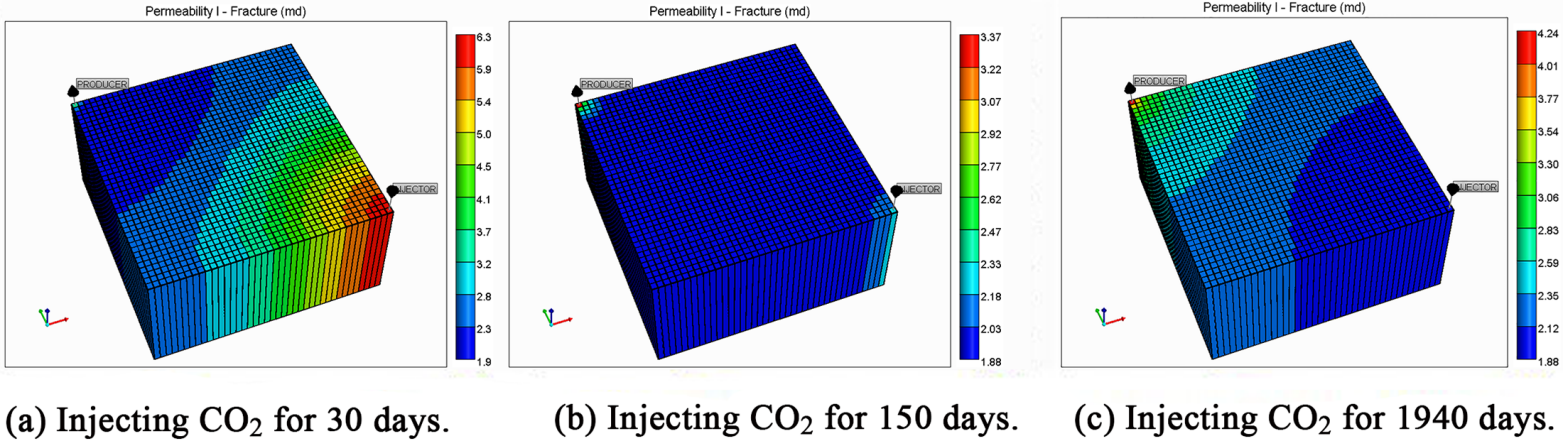
Permeability distribution in the reservoir considering both matrix shrinkage and cleat deformation.

**Fig 4:**
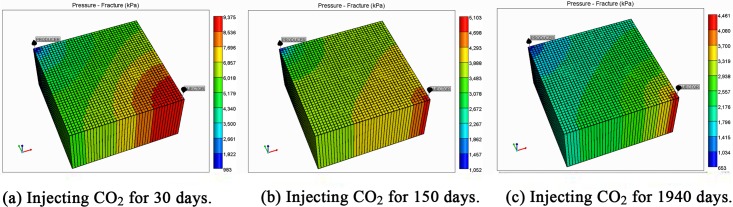
Pressure distribution in the reservoir considering both matrix shrinkage and cleat deformation.

To validate the effects of matrix shrinkage on fracture permeability, two different permeability equations were used in the simulation. One considers both cleat deformation and matrix shrinkage, and the other considers only cleat deformation. Fig 3 shows the permeability distribution in the reservoir at various simulation times under conditions of both matrix shrinkage and crack deformation. To improve calculation speed and save calculation time, the grids used in the simulation are limited. This results in distribution contours that are not very smooth, but it does not affect the accuracy of the simulation. Because the permeability changes are affected by reservoir pressure, Fig 4 shows the pressure distribution of the reservoir at various simulation times.

**Fig 5:**
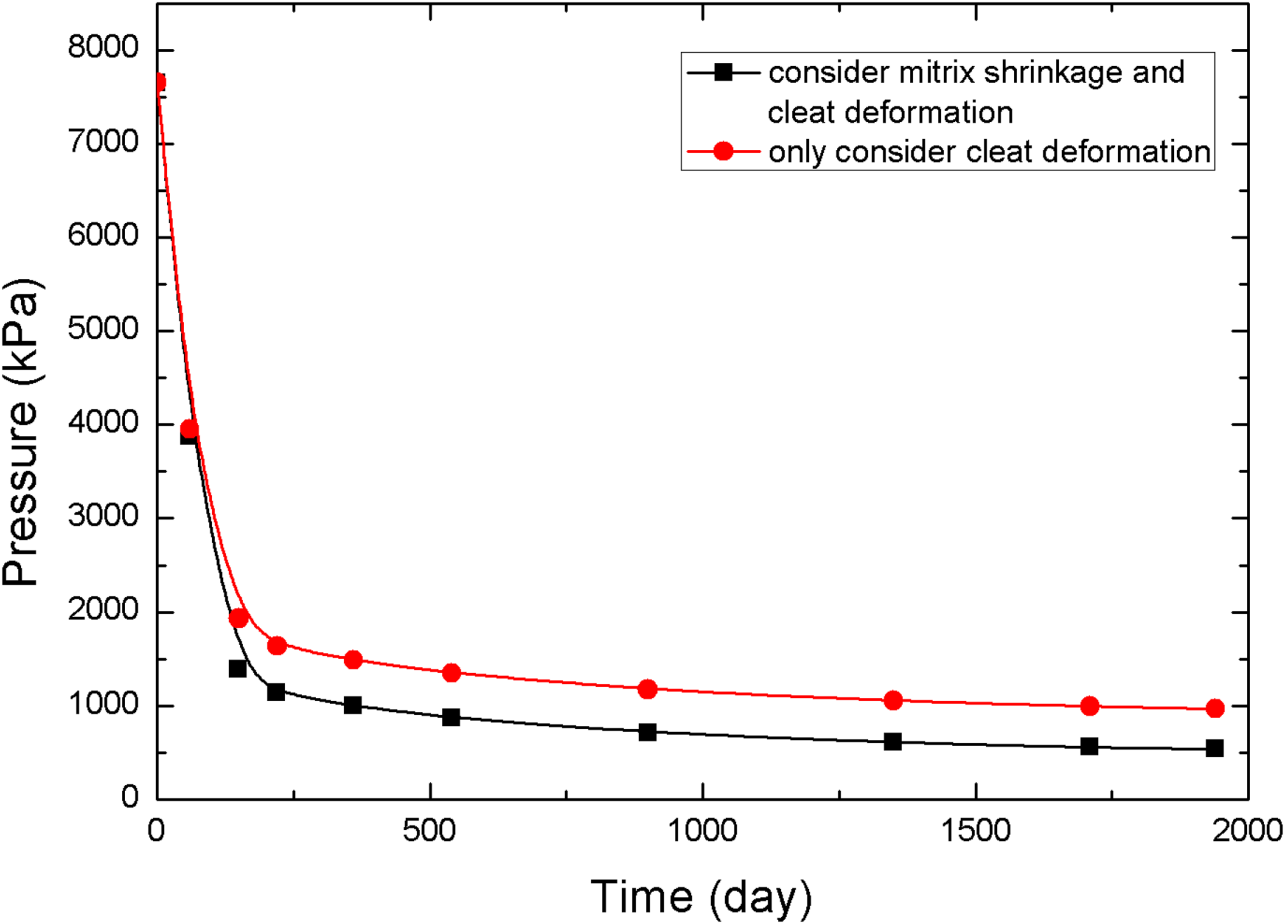
Pressure change curve at point 1.

**Fig 6:**
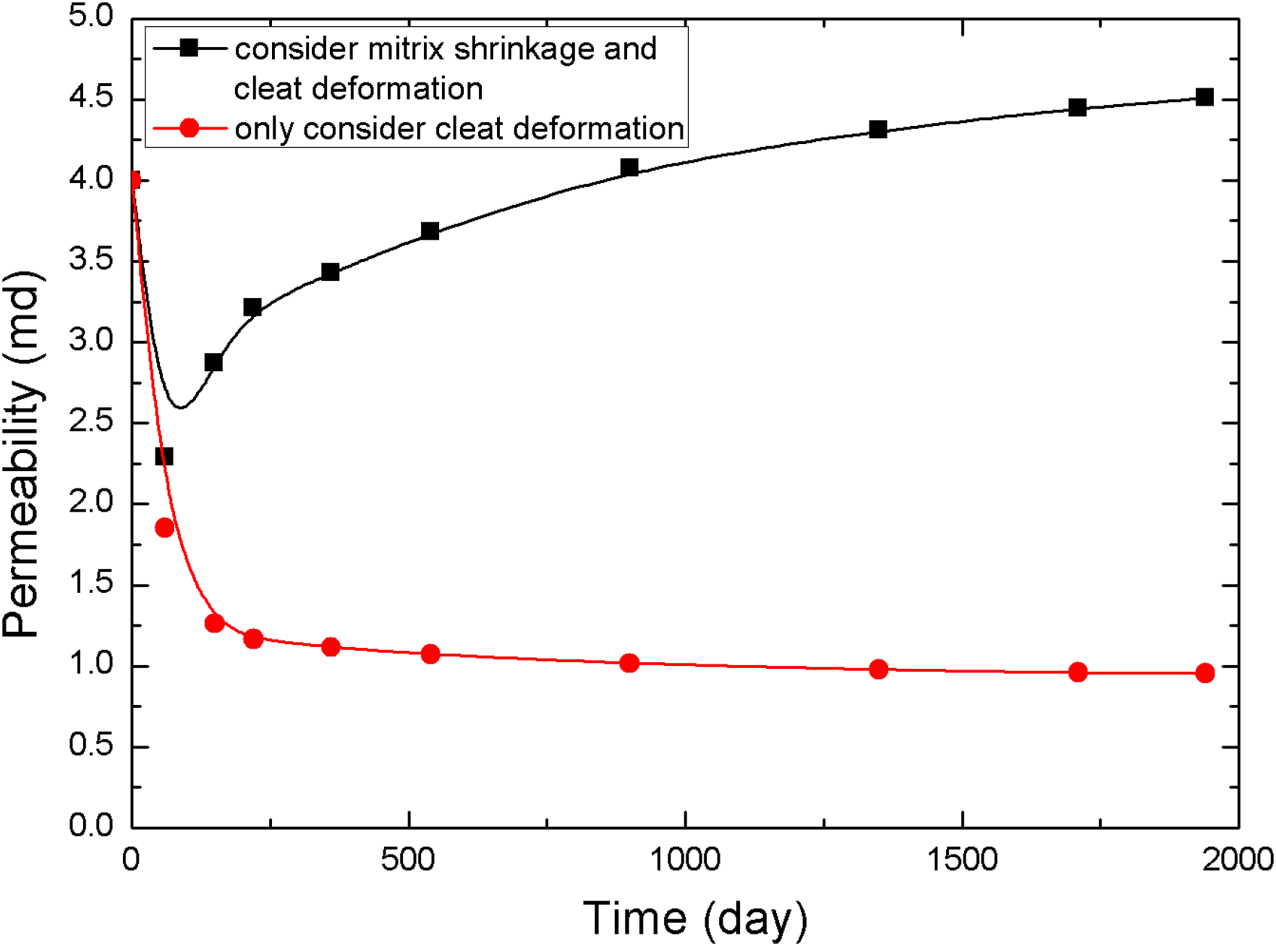
Permeability change curve at point 1.

**Fig 7:**
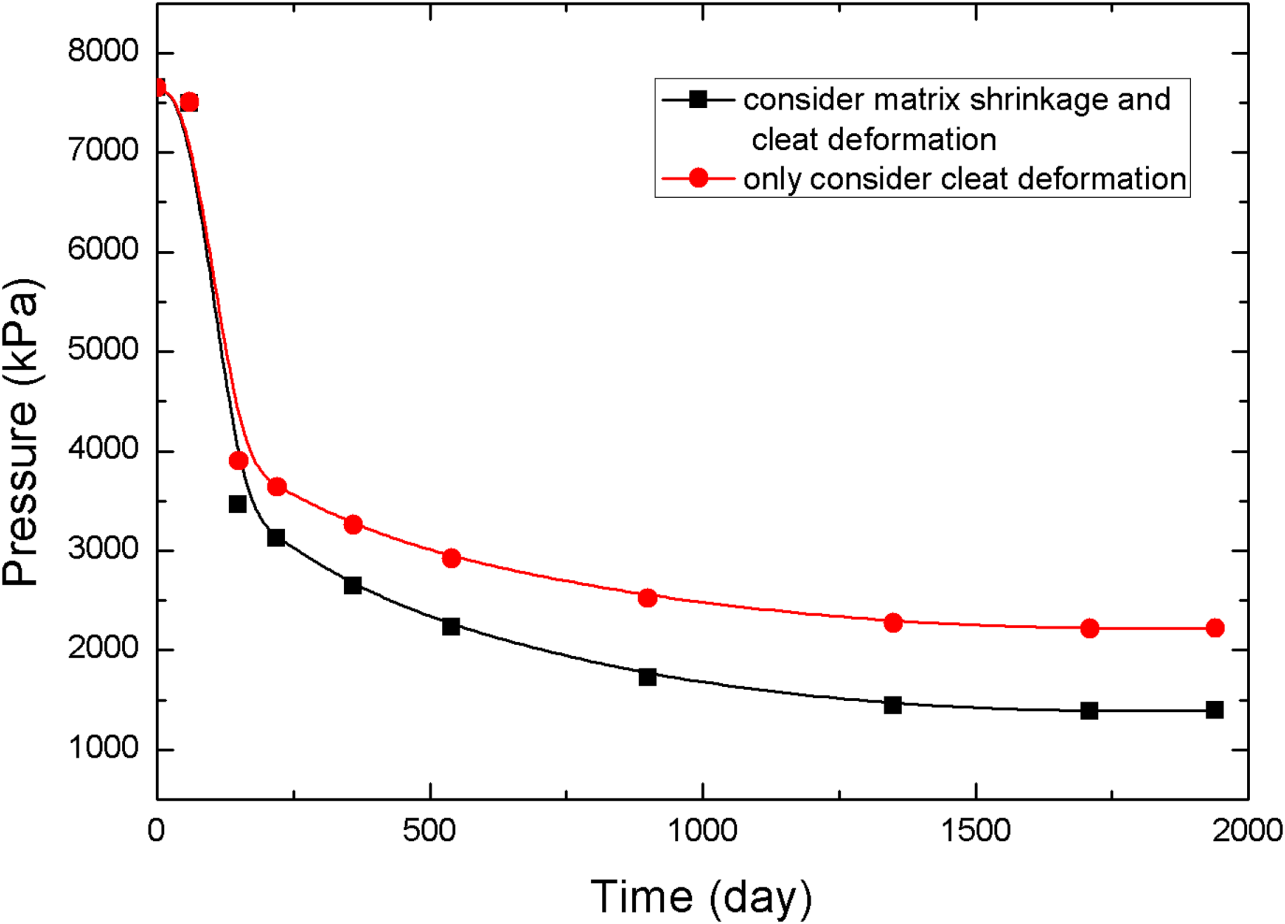
Pressure change curve at point 2.

**Fig 8:**
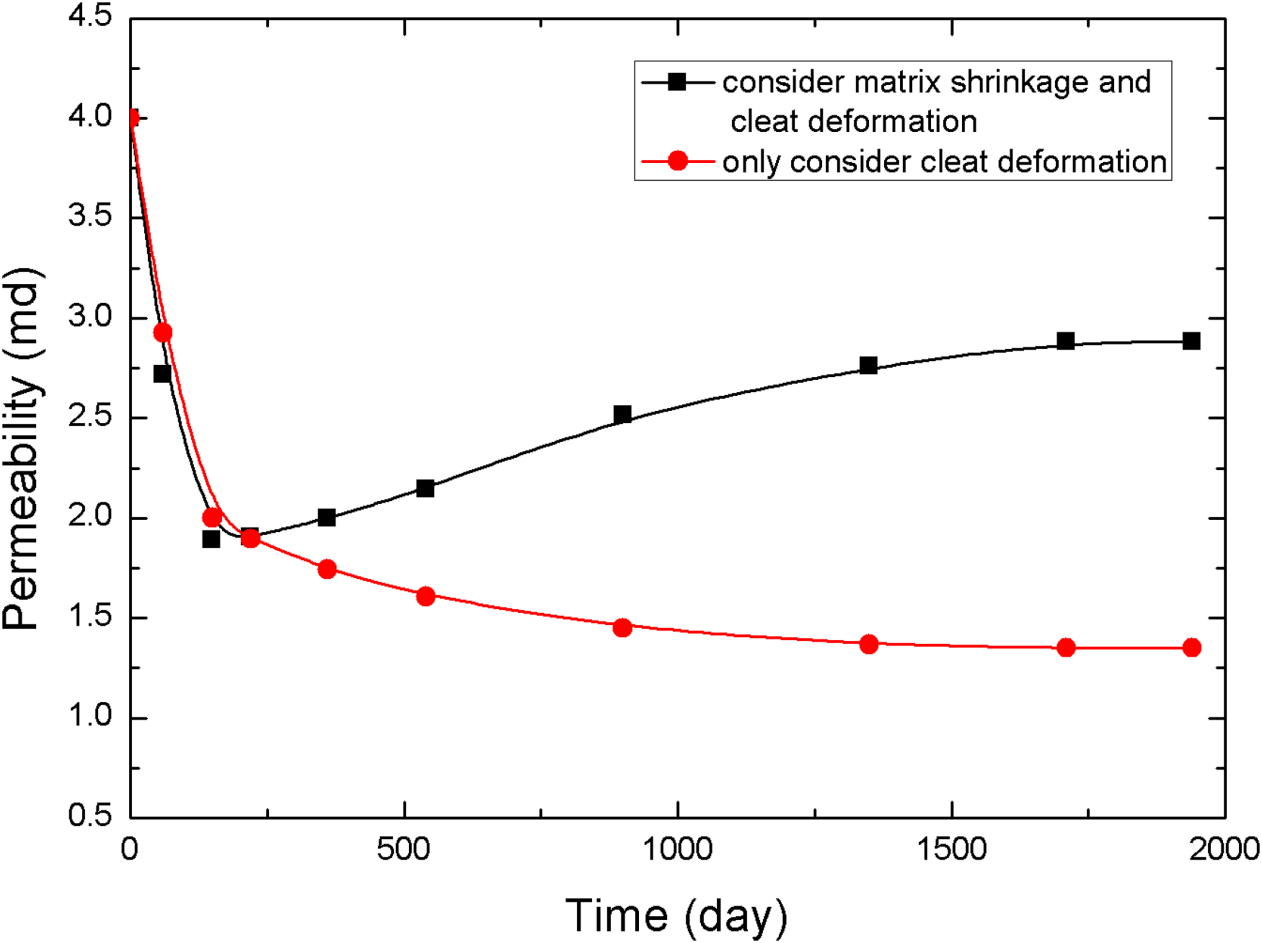
Permeability change curve at point 2.

**Fig 9:**
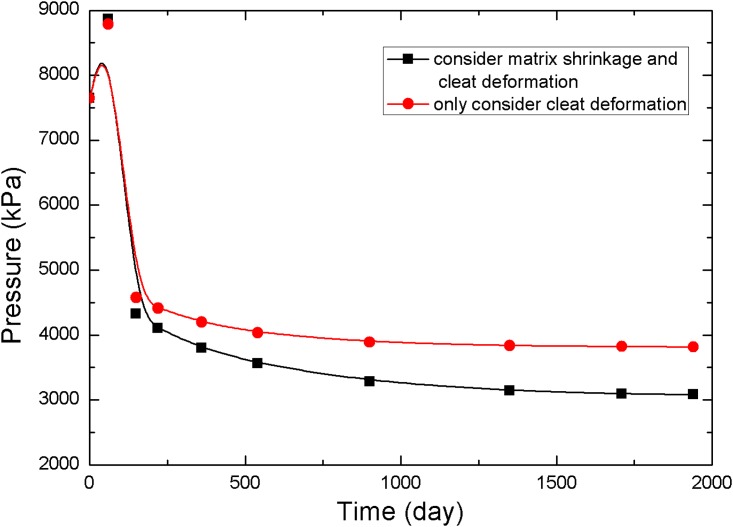
Pressure change curve at point 3.

**Fig 10:**
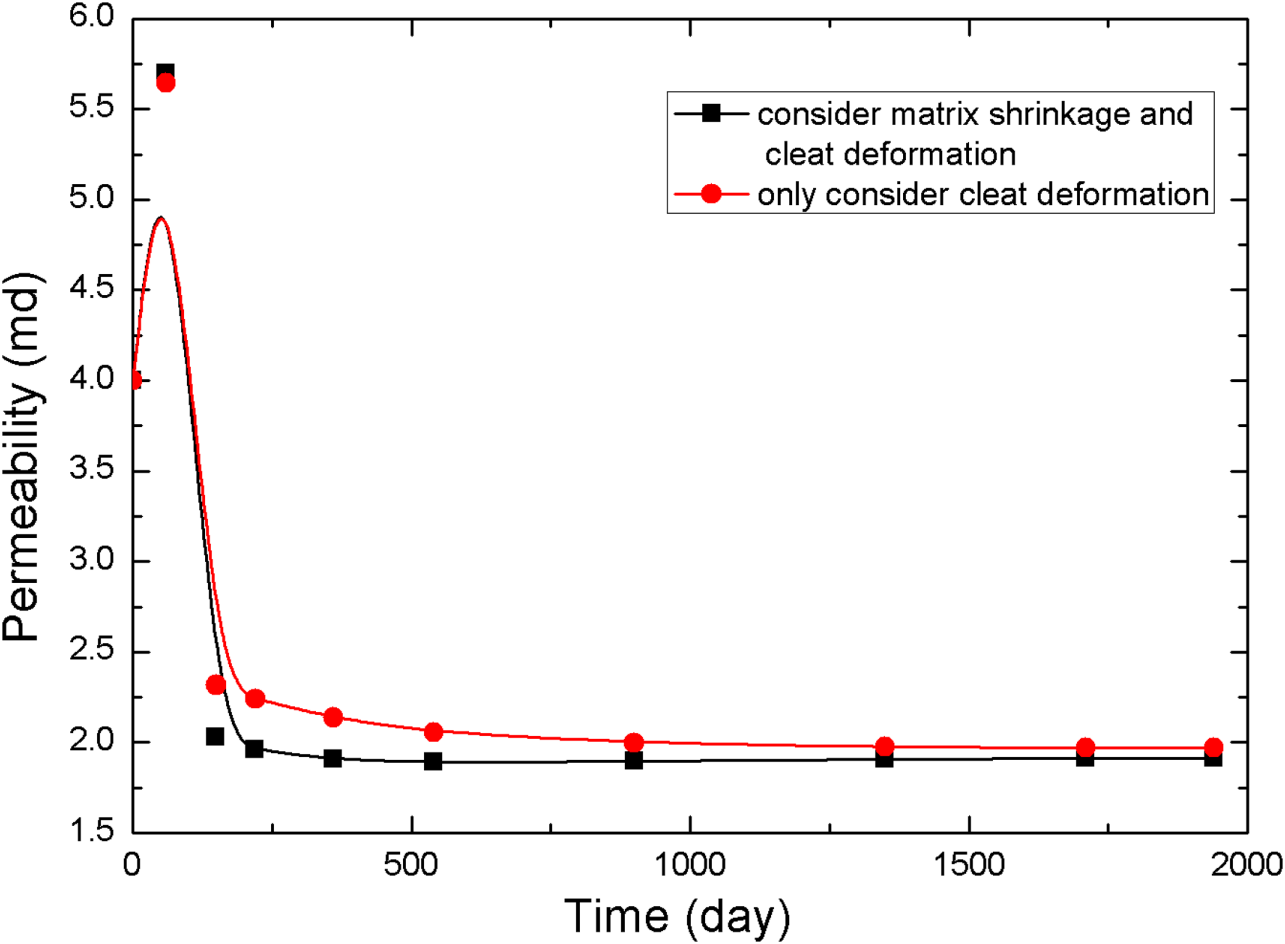
Permeability change curve at point 3.

The points 1, 2, and 3 in Fig 1 (point 1 is near the production well, point 2 is in the middle of the reservoir, and point 3 is near the injection well) are selected to analyze the pressure and permeability data at different times, resulting in the curves in Figs 5–10.

The value of permeability at point 1 during the whole simulation period is shown in Fig 6. At the beginning of the simulation, the reservoir pressure near the wellbore rapidly decreases to less than 3.5 MPa (Fig 5), and the permeability near the wellbore appears to rebound due to matrix shrinkage. At low drawdown pressures, the degree of permeability rebound is greater. One interpretation of this pattern is that matrix shrinkage increases pore volume, leading to an increase in permeability. In a simulation of 1,940 days, the maximum value of permeability around the production well increased to 4.3 mD, exceeding the initial permeability in the reservoir. Fig 6 shows a typical permeability curve near the production well at various periods when permeability is influenced only by cleat deformation. As the output of CH_4_ and pore pressure decrease, the effective stress increases and cracks are compressed. This process makes the fluid pathways smaller and leads to a decrease in permeability.

Fig 8 presents the changes in permeability in the middle of the reservoir at various times. Because the pressure decrease was weaker than that of the area near the production well, the permeability rebound was not large, and the final value was less than 3 mD. However, the permeability still increased by 1.5 mD more than the permeability in the case in which the fracture permeability formula does not consider matrix shrinkage.

As a result of CO_2_ injection, the pressure of the reservoir was replenished, slowing the pressure decrease in the reservoir, especially near the injection well where the pressure was maintained at a high value (Fig 4). The permeability in these areas consistently decreased, and the permeability curve was similar to the case that only considers cleat deformation (Fig 10).

**Fig 11:**
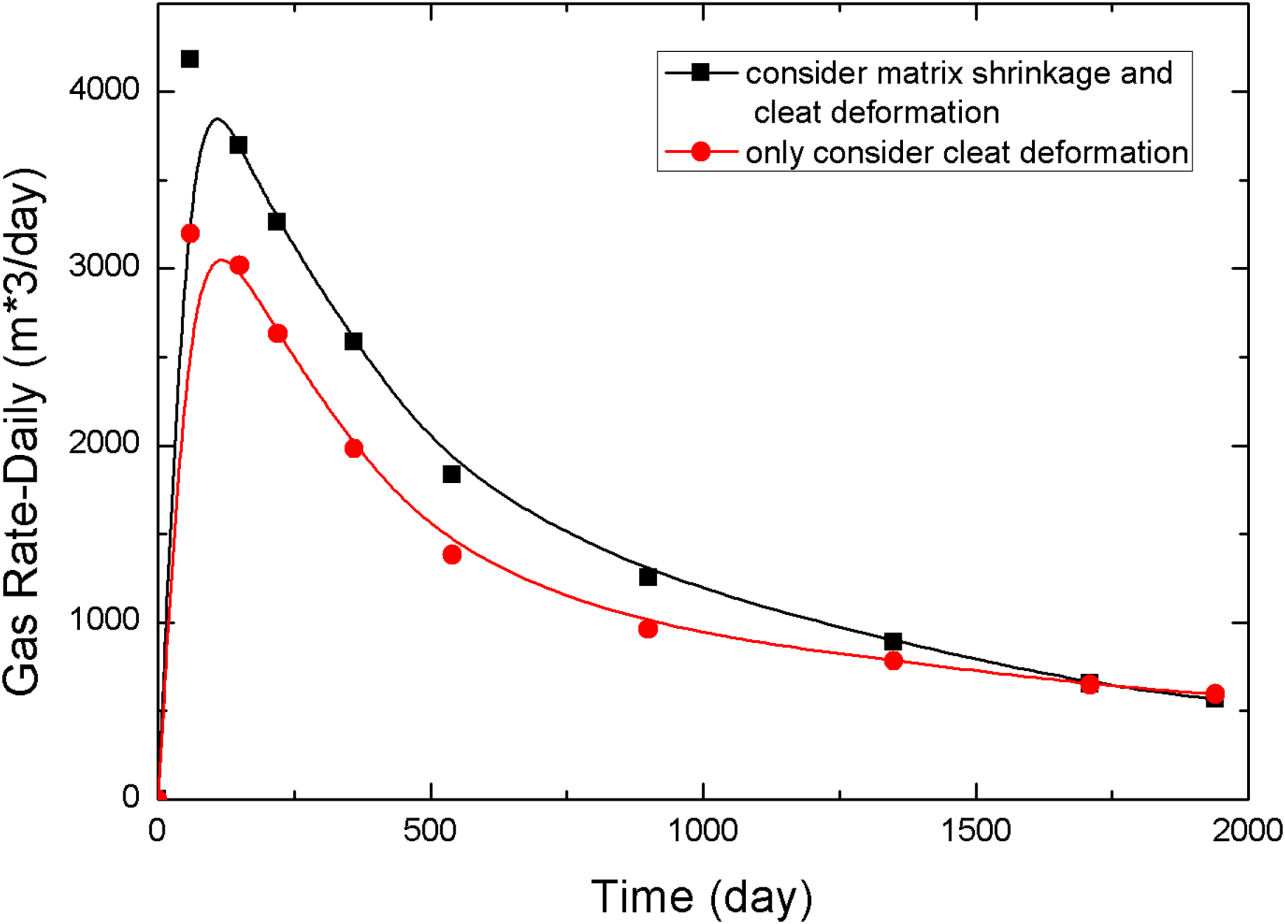
Comparison of daily gas rate.

Fig 11 compares the daily gas rates of both cases to analyze the effects of matrix shrinkage on CO_2_ flooding of coalbed methane (CO_2_-ECBM). The peak value of daily gas rate was higher when matrix shrinkage is considered, resulting in a maximum value of 4182 m^3^/day, which is 30.77% higher than that of the case that only considers cleat deformation. In the late stage of the simulation, low gas saturation becomes the main factor limiting the daily gas rate. After a simulation length of 1,700 days, the daily gas rate drops below that of the case that only considers cleat deformation. Therefore, matrix shrinkage improves the fracture permeability, thereby increasing CH_4_ production during the early stages and decreasing gas saturation during the later stages.

**Fig 12:**
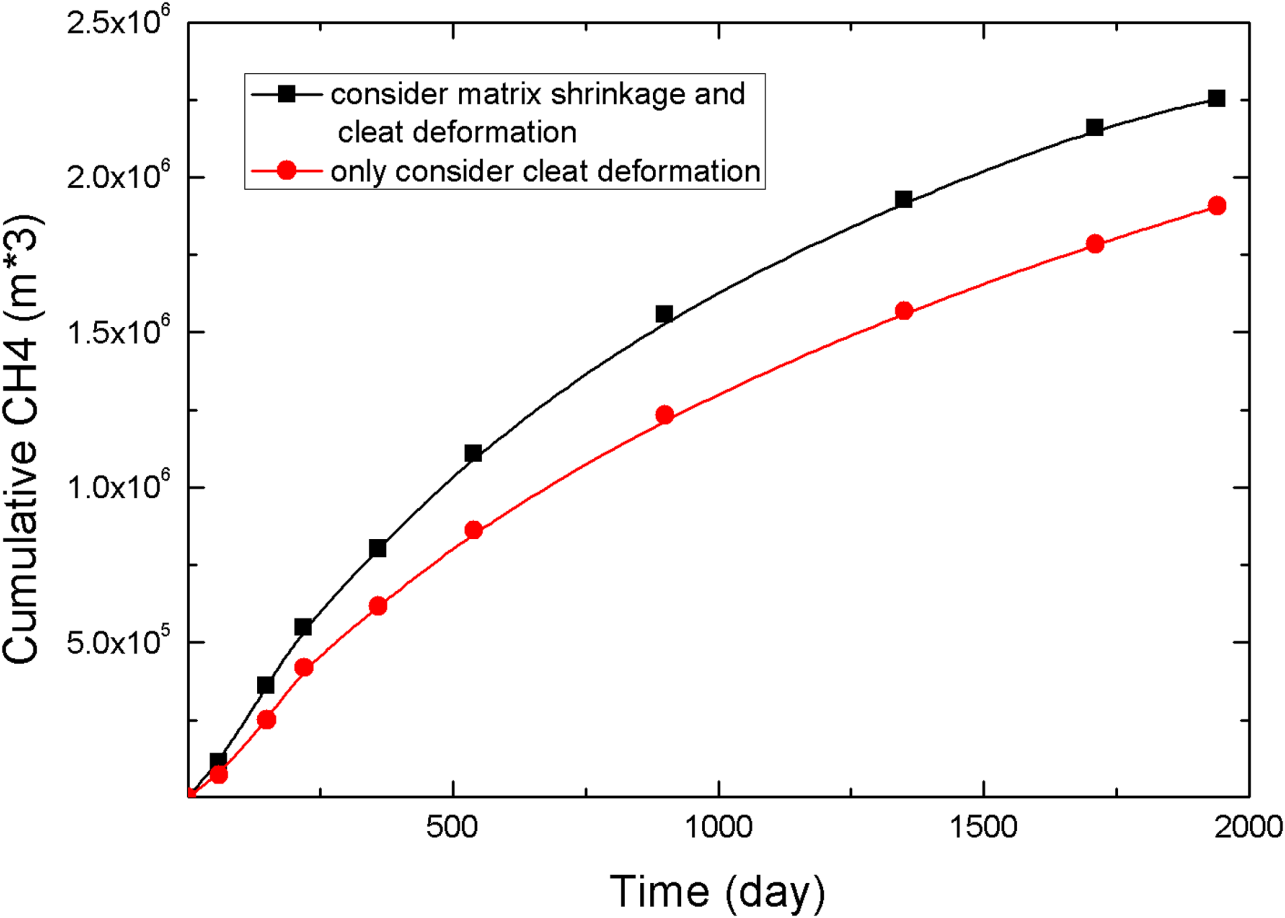
Comparison of cumulative production.

The evolution of the cumulative production of CH_4_ is shown in Fig 12. The curve clearly indicates that matrix shrinkage promotes the CO_2_-ECBM process. Matrix shrinkage allows fracture permeability to increase and accelerates the output of gas. Under the given conditions in this study, the cumulative production of CH_4_ was 2.25×10^6^ m^3^, and the recovery was 80.3%, which was 18.1% higher than the case that did not consider matrix shrinkage.

## Conclusions

A coupled mathematical model of CO_2_ flooding that considers coal or rock deformation and multi-physical processes (competitive adsorption, convection-diffusion, seepage) was established in this paper. Using the simulation software to solve the coupling model, our study emphasized the influence of coal matrix shrinkage on permeability during CO_2_ flooding. The conclusions have been reached:

1. Based on the initial pressure and the differences in pressure variations during the production process, the permeability changes caused by matrix shrinkage are spatially variable in the reservoir. The maximum permeability value appears near the production well, and the degree of rebound decreases with increasing distance from the production well.
2. Under the conditions of our study, matrix shrinkage has an galvanizing effect on CO_2_-ECBM and increases the daily gas rate during the early production phase. Although the CH_4_ saturation is lower in later stages, resulting in lower daily gas rates, the overall final yield is greater.
3. In general, permeability in coals is a function of pressure drawdown. In the CO_2_-ECBM process, CO_2_ injection changes the distribution of pressure, which changes the permeability in the reservoir. Therefore, selecting the appropriate well spacing and injection rate based on different reservoir characteristics is necessary to ensure that the CO_2_ injection will not reduce the permeability of the reservoir and to achieve the optimal effects of CO_2_ displacement.
